# Statins Promote the Regression of Atherosclerosis via Activation of the CCR7-Dependent Emigration Pathway in Macrophages

**DOI:** 10.1371/journal.pone.0028534

**Published:** 2011-12-06

**Authors:** Jonathan E. Feig, Yueting Shang, Noemi Rotllan, Yuliya Vengrenyuk, Chaowei Wu, Raanan Shamir, Ines Pineda Torra, Carlos Fernandez-Hernando, Edward A. Fisher, Michael J. Garabedian

**Affiliations:** 1 Division of Cardiology (Marc and Ruti Bell Program in Vascular Biology), Department of Medicine, New York University School of Medicine, New York, New York, United States of America; 2 Department of Microbiology, New York University School of Medicine, New York, New York, United States of America; Ulm University, Germany

## Abstract

HMG-CoA reductase inhibitors (statins) decrease atherosclerosis by lowering low-density-lipoprotein cholesterol. Statins are also thought to have additional anti-atherogenic properties, yet defining these non-conventional modes of statin action remains incomplete. We have previously developed a novel mouse transplant model of atherosclerosis regression in which aortic segments from diseased donors are placed into normolipidemic recipients. With this model, we demonstrated the rapid loss of CD68+ cells (mainly macrophages) in plaques through the induction of a chemokine receptor CCR7-dependent emigration process. Because the human and mouse CCR7 promoter contain Sterol Response Elements (SREs), we hypothesized that Sterol Regulatory Element Binding Proteins (SREBPs) are involved in increasing CCR7 expression and through this mechanism, statins would promote CD68+ cell emigration from plaques. We examined whether statin activation of the SREBP pathway *in vivo* would induce CCR7 expression and promote macrophage emigration from plaques. We found that western diet-fed apoE^-/-^ mice treated with either atorvastatin or rosuvastatin led to a substantial reduction in the CD68+ cell content in the plaques despite continued hyperlipidemia. We also observed a significant increase in CCR7 mRNA in CD68+ cells from both the atorvastatin and rosuvastatin treated mice associated with emigration of CD68+ cells from plaques. Importantly, CCR7^-/-^/apoE^-/-^ double knockout mice failed to display a reduction in CD68+ cell content upon statin treatment. Statins also affected the recruitment of transcriptional regulatory proteins and the organization of the chromatin at the CCR7 promoter to increase the transcriptional activity. Statins promote the beneficial remodeling of plaques in diseased mouse arteries through the stimulation of the CCR7 emigration pathway in macrophages. Therefore, statins may exhibit some of their clinical benefits by not only retarding the progression of atherosclerosis, but also accelerating its regression.

## Introduction

Atherosclerosis is responsible for more than half of all mortality in Western countries. Elevated low-density-lipoprotein cholesterol (LDL-C) is an established risk factor for coronary artery disease. Inhibitors of 3-hydroxy-3-methylglutaryl coenzyme A (HMG-CoA) reductase, statins, are lipid-lowering drugs that effectively lower LDL-C level and reduce the risk of cardiovascular events in hypercholesterolemic and normocholesterolemic patients [1]. Clinical studies also suggest that statins may exert vasculoprotective effects that are independent of their cholesterol-lowering properties. Pleiotropic effects of statins include the improvement of endothelial function and reduction in oxidative stress, inhibition of inflammation, and stabilization of atherosclerotic plaques [2], [3], [4].

As useful as statins may be in limiting progression of cardiovascular disease, there is likely to be a significant plaque burden remaining in the treated population. In spite of the clinical desirability to achieve regression and the success of statin treatment to achieve it in some patients [5], [6], research into the factors that may be mediating this process has been hampered by the relative paucity of appropriate animal models. The similarities between atherosclerosis progression in humans and mice deficient either in apoE (apoE^-/-^) or the LDL receptor suggest that molecular mechanisms underlying regression in these mouse models could be relevant to the reduction in plaque burden in the human population (reviewed in [3], [7]).

Regression studies in mice, indeed, have been undertaken, with some modest successes reported (reviewed in [4]). To introduce a more robust model, we developed an approach in which transplantation of either an atherosclerotic-containing thoracic aortic segment [8] or an aortic arch segment [9] from apoE^-/-^ mice to wild-type (WT) recipient mice leads to the dyslipidemia being corrected indefinitely. Under the conditions of the WT mouse, regression is rapidly apparent (as judged by plaque content of cells positive for CD68, an accepted marker of macrophages and macrophage-foam cells), whereas when the recipient is an apoE^-/-^ mouse, further progression is evident [10], [11], [12].

Notably, the decrease in CD68+ cell content could be attributed to emigration of these cells from plaques to regional and systemic lymph nodes under regression, but not progression, conditions [11], [12]. The emigrating cells expressed markers of dendritic cells (DCs), which like macrophages, can derive from monocytes [13]. Because migration of DCs to lymph nodes absolutely requires the chemokine receptor CCR7 [14], we hypothesized that it became induced in CD68+ cells under regression conditions. Indeed, we found an increase in CCR7 mRNA and protein expression only in plaque CD68+ cells from the regression environment and went on to show the functional requirement of CCR7 for regression in our transplant model [12], [15].

The importance of this gene has led us to study its regulation. Interestingly, bioinformatic analysis revealed putative sterol response elements (SREs) along the promoter region of CCR7. The presence of such elements suggest that sterol response element binding proteins (SREBPs)[16] may bind to those sites and that through this molecular mechanism, statins may regulate CCR7 expression. Given these considerations, we examined whether statin therapy provides an additional benefit to the arterial wall by promoting regression through this mechanism in a mouse model of atherosclerosis. Our findings from both studies *in vitro* and *in vivo* suggest that this is indeed the case.

## Results

### Atorvastatin and rosuvastatin decrease total cholesterol without significantly affecting HDL-C in western-diet fed apoE^-/-^ mice

To examine the effects of atorvastatin and rosuvastatin on total cholesterol levels, apoE^-/-^ mice were fed an atherogenic “western diet” for 16 weeks and then fed for 4 weeks a western diet containing either atorvastatin or rosuvastatin milled into the food. A group of mice not treated with statins, but maintained on the western diet, served as the baseline control group. Total cholesterol and high density-lipoprotein cholesterol (HDL-C) from serum were measured for each group. As expected, in the baseline untreated group, total cholesterol levels were extremely high (1313 mg/dL; normal ∼80 mg/dL), whereas the atorvastatin and rosuvastatin treated groups had lower total cholesterol levels of 767 mg/dL and 593 mg/dL, respectively (Table 1). Although both statins were able to significantly reduce total cholesterol levels, the atorvastatin and rosuvastatin treated mice remained significantly hyperlipidemic, maintaining levels known to promote atherosclerosis progression [7], [17]. Importantly, though the atorvastatin and rosuvastatin treated group reduced the total cholesterol by roughly half of that of the control group, HDL-C levels were not significantly changed (Table 1). Thus, both atorvastatin and rosuvastatin were capable of lowering total cholesterol levels without affecting HDL-C, and at the doses used, rosuvastatin appeared to be more effective in lowering total cholesterol than atorvastatin.

**Table 1:** Statin treatment decreases total cholesterol without affecting HDL-C in western-diet fed apoE^-/-^ mice.

	Baseline	Atorvastatin	Rosuvastatin
**Total Cholesterol (mg/dL)**	**1313±138**	**767±40**	**593±31**
**HDL Cholesterol (mg/dL)**	**30.8±3.3**	**32.3±6.0**	**36.2±2.8**

Total cholesterol and HDL cholesterol levels were measured (n = 10 mice per group). Listed are the average values with standard errors of the means.

### Statin treatment decreases the contents of CD68+ cells and cholesteryl ester in atherosclerotic plaques while increasing collagen deposition

**Figure 1:**
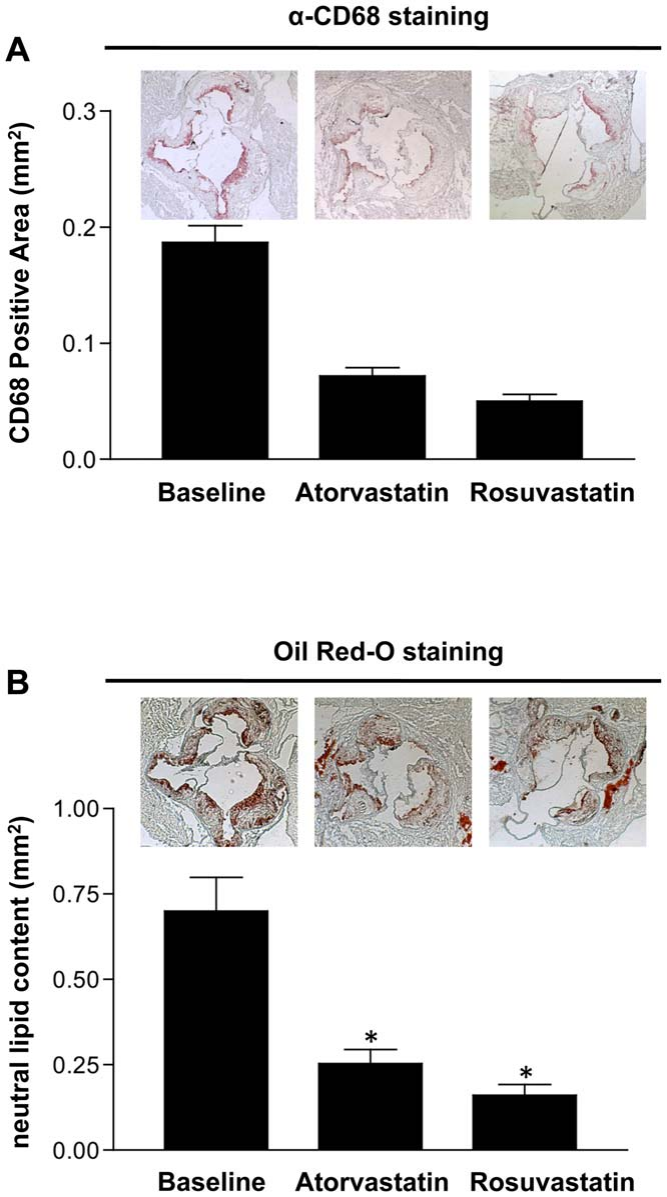
Statin treatment decreases CD68+ cell and cholesteryl ester contents in atherosclerotic plaques. ApoE^-/-^ mice (n = 10 per group) were fed a high fat diet (western diet) after which they were either maintained on the western diet, or switched to a western diet that included atorvastatin or rosuvastatin. A) Macrophage content was assessed by CD68 staining, and B) cholesteryl ester content was assessed by oil red-O staining. The symbol * indicates statistical significance, p<0.05.

We next examined the impact of statin treatment on plaque CD68+ cell and cholesterol ester content. To measure CD68+ cell content, frozen sections were immunostained and the positive areas were quantified by computer-aided morphometric analysis. Over 65% and 75% of the CD68+ cell contents were reduced in the atorvastatin and rosuvastatin treated groups, respectively, compared to baseline controls (Figure 1A). Interestingly, neutral lipid content (expected to be primarily cholesteryl esters), as measured by oil red-O staining, was also decreased by roughly 60% in both treatment groups (Figure 1B). Thus, both atorvastatin and rosuvastatin reduced plaque CD68+ cell and cholesterol ester content *in vivo*.

**Figure 2:**
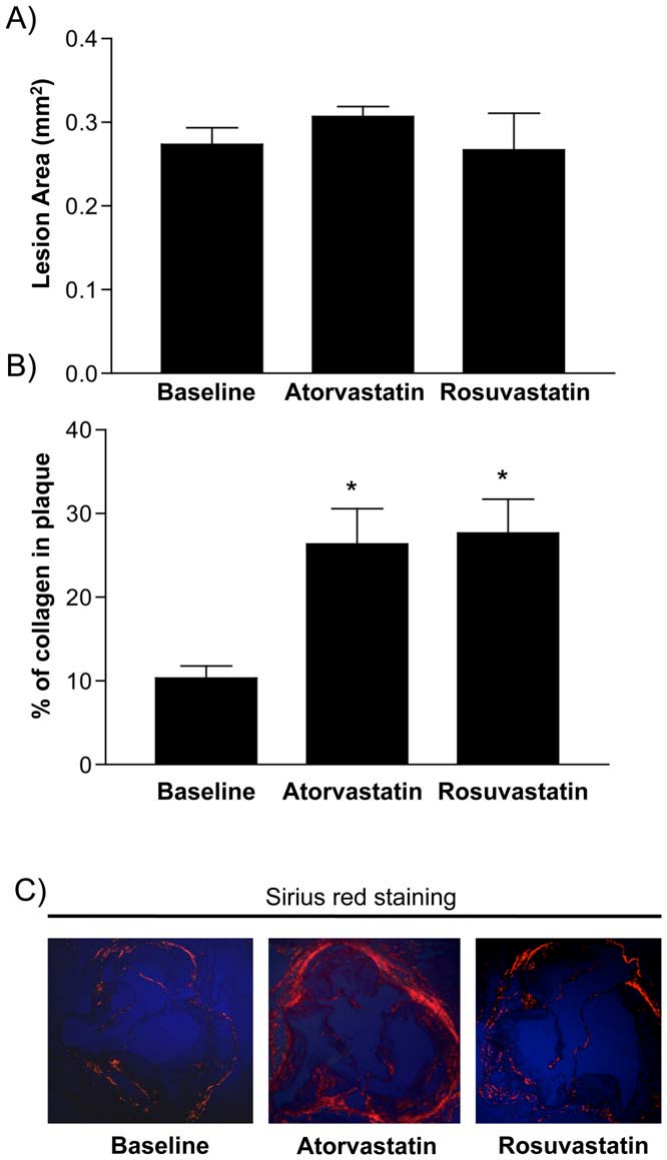
Statin treatment does not significantly affect plaque area likely due to the increase in collagen content. A) Plaque size was assessed by morphometric analysis after hematoxylin and eosin staining of serial sections (n = 10 per group). B) Collagen levels were measured using Sirius red staining under polarizing light microscopy (n = 10 per group). The symbol * indicates statistical significance, p<0.05.

Although CD68+ cell content significantly decreased in both statin-treated groups, plaque size appeared not to significantly change (Figure 2A). This suggested that other plaque components, such as collagen, might have increased. Using Sirius red staining, we found a significant increase in collagen content in plaques from the statin-treated mice as compared to controls (Figure 2B and C).

### Statin treatment promotes monocyte emigration out of atherosclerotic plaques in a CCR7-dependent manner

We previously reported that regression is characterized by emigration of monocyte-derived cells out of atherosclerotic lesions [11], [12]. There are two major subsets of monocytes expressing chemokine receptors to different degrees: 1) Ly-6C^hi^ that express both CX3CR1 (fractalkine receptor) and CCR2 (chemokine receptor 2), with higher expression of CCR2; and 2) Ly-6C^lo^ that express high levels of CX3CR1, but low levels of CCR2. Given that both CCR2 and CX3CR1 have been linked to progression of atherosclerotic plaques (e.g.,[18]), we asked whether statin treatment could promote the migration of these subsets out of plaques.

**Figure 3:**
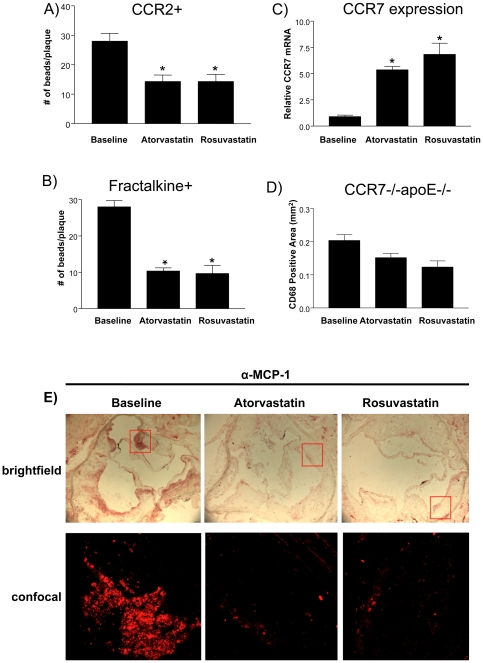
Statin treatment promotes Ly-6C^hi^ and Ly-6C^lo^ monocyte emigration from atherosclerotic plaques in a CCR7-dependent manner and represses MCP-1 expression. The number of beads remaining in the plaque 4 weeks post-statin treatment that corresponded to; A) CCR2 (Ly-6C^hi^) monocytes and B) CX_3_CR1 (Ly-6C^lo^) highly positive monocytes are shown (n = 8 per group). C) Relative CCR7 mRNA expression levels as a function of statin treatment were determined by RT-PCR (n = 9 per group). D) CD68+ cell content in plaques from CCR7^-/-^apoE^-/-^ double knockout mice treated with statin were determined by morphometric analysis of the immunostained areas (n = 7 per group). The symbol * indicates statistical significance, p<0.05. E) MCP-1 expression in plaques as a function of statin treatment. Sections of aortic roots from baseline, atorvastatin and rosuvastatin treated mice were stained with a biotinlyated mouse MCP-1 antibody, and visualized using chromogenic and fluorescent reaction products. Brightfield images of MCP-1 immunostaining of the whole root (top row) and confocal fluorescent images of the selected areas (inserts, bottom row) are shown and demonstrate reduced levels of MCP-1 protein in plaques of statin treated mice compared to baseline controls.

The ability to differentially label and track these monocyte subsets has been recently reported [19]. Applying these methods to our mice, we found that the content of cells derived from either subset of monocyte decreased in plaques after 4 weeks of statin treatment (Figures 3A and B). These results supported a role for statins in inducing cells of monocyte origin to migrate from an atherosclerotic plaque.

We next addressed the mechanism of statin-induced monocyte emigration. We previously reported in a transplant-based mouse model of atherosclerosis regression that the rapid depletion of plaque CD68+ cells was through a migration process dependent upon the induction of chemokine receptor CCR7 [12]. To determine whether a similar mechanism was operating in statin-treated mice, we measured CCR7 mRNA from laser-captured CD68+ cells from these and control mice. We found that CCR7 mRNA was increased in both the atorvastatin and rosuvastatin treated mice more than 5X (p<0.05) compared to baseline control mice (Figure 3C).

To directly test the requirement for CCR7 in statin induced foam cell depletion, we generated CCR7^-/-^/apoE^-/-^ double knock out mice, placed them on a western diet and switched half to the statin-treated western diet for 4 weeks. Importantly, plaque CD68+ cell depletion in the CCR7^-/-^ mice was significantly impaired in the statin treated mice as compared to the baseline controls. This strongly suggests that CD68+ cell migration induced in plaques by statins is CCR7 dependent (Figure 3D).

We also examined the levels of the proinflammatory chemokine monocyte chemoattractant protein-1 (MCP-1). We find a decrease in MCP-1 protein expression in both the atorvastatin and rosuvastatin-treated mice compared to baseline control mice (Figure 3E). This suggests the statin treatment promotes a more anti-inflammatory environment in the plaques, as has been reported before [20].

### Statins induce SREBP regulation of CCR7 expression

**Figure 4:**
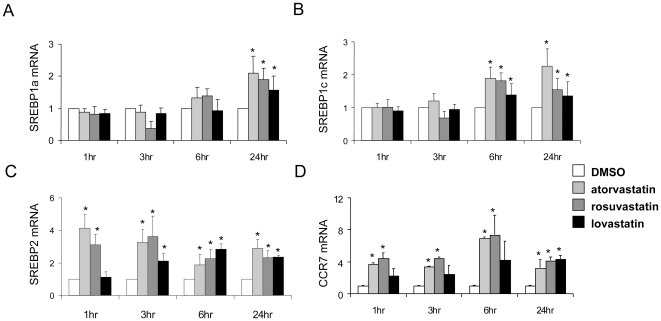
Statins Induce SREBP and CCR7 expression. RAW264.7 macrophage cells were treated with 5 μM statins for the times indicated, and expression of SREBP-1a (A), SREBP-1c (B), SREBP-2 (C), and CCR7 (D) were determined by RT-PCR. The experiments were repeated 3 times with similar results. The symbol * indicates statistical significance, p<0.05.

The above results imply that statins can modulate CCR7 expression and function. We analyzed *in silico* the CCR7 gene from human and mouse for common motifs and response elements and identified consensus sterol binding protein response elements (SRE) at ∼220 bp upstream of the transcription start site (Figure S1). Given that statins increase the activation of SRE binding proteins (SREBPs) [21], we first examined whether the three SREBP isoforms, SREBP-1a, SREBP-1c, and SREBP-2, were induced at the mRNA level by statins. Atorvastatin, rosuvastatin, and lovastatin all induced these three isoforms in RAW264.7 macrophages (a mouse cell line) by 24 hours (Figure 4A-C). Only SREBP-2 was induced in as little as one-hour post statin treatment and remained up regulated throughout the 24-hour time-point (Figure 4C). CCR7 gene expression was also induced by statins at 1 hour with maximal induction at the 6 hour time point, with the greatest induction by rosuvastatin (Figure 4D). Therefore, the subsequent experiments were performed with rosuvastatin. Given the rapid and robust induction of SREBP-2, we hypothesized that it regulates CCR7 expression in macrophages. Consistent with this was that of the three SREBP isoforms only SREBP-2 was significantly induced in laser-captured CD68+ cells from atherosclerotic plaques in statin-treated mice (not shown).

### Statins regulate CCR7 expression through modulating SREBP-2

**Figure 5:**
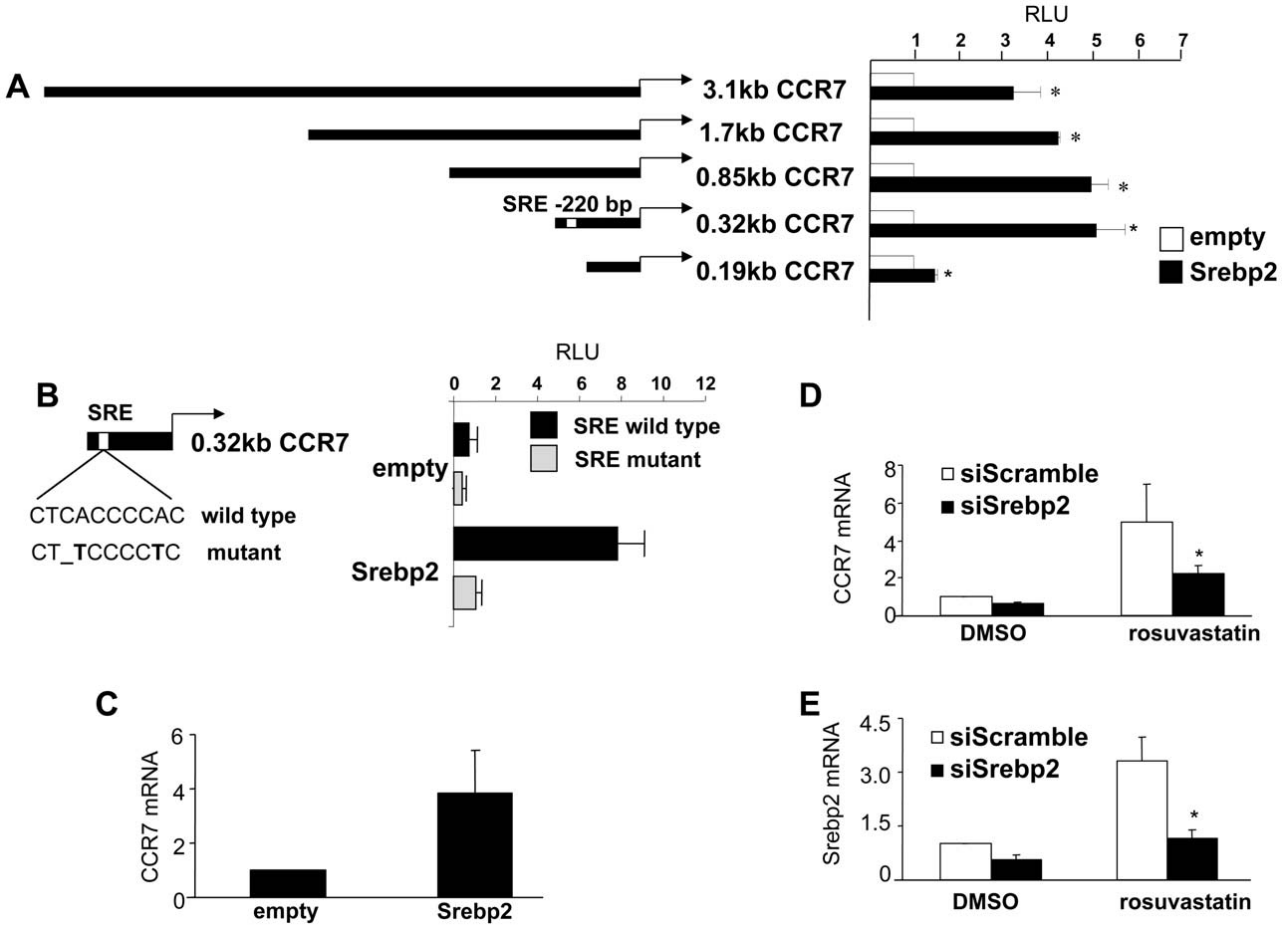
Statins regulate CCR7 expression through modulating SREBP-2. A) Luciferase assays were performed in RAW264.7 cells co-transfected with expression plasmids for SREBP-2 and a series of CCR7 reporter constructs in the absence (DMSO) and presence of rosuvastatin treatment. Relative luciferase units (RLU) normalized to β-galactosidase activity are shown. B) The consensus SRE-binding site in the -0.32 kb CCR7-luciferase reporter was mutated as indicated. COS7 cells were co-transfected with an empty vector (pCDNA3) or mature SREBP-2, along with either the wild type or mutated SRE-reporter construct and RLU measured as above. C) RAW264.7 cells were transfected with an SREBP-2 expression plasmid or control empty vector control, and expression of CCR7 was determined by RT-PCR. D) SREBP-2 and E) CCR7 mRNA expression was measured by RT-PCR after siRNA knockdown with a scrambled siRNA control or a specific siRNA against SREBP-2 in the absence and presence of 5 µM rosuvastatin. The symbol * indicates statistical significance, p<0.05.

To demonstrate that the CCR7 promoter has a functional SRE *in vitro*, RAW 264.7 cells were co-transfected with a series of deletion constructs spanning the 5′ upstream region of the mouse CCR7 gene and an expression vector for SREBP-2. Each CCR7 promoter sequence was linked to a luciferase reporter gene, and luciferase activity was measured to estimate promoter activity. As can be seen in Figure 5A, CCR7 5′-truncations from -3100 to -320 did not compromise SREBP-2 induction of CCR7 expression. In contrast, a deletion from -320 to -190 reduced CCR7 promoter activity, suggesting that elements required for SREBP-2-dependent CCR7 expression lie between -320 and -190bp, which coincides with the predicted location of the SRE in CCR7.

The importance of the SRE for CCR7 promoter activity was assessed using CCR7-luciferase reporter constructs with point mutations in the SRE element (Figure 5B). Mutation of the SRE compromised CCR7 promoter activity by more than 80% upon expression of mature SREBP-2 (Figure 5B), suggesting that the SRE binding site is important for SREBP-2-dependent CCR7 promoter activity.

Additionally, we were able to demonstrate that over-expression of SREBP-2 can increase the expression of endogenous CCR7 mRNA in RAW264.7 cells (Figure 5C). Furthermore, when SREBP-2 was silenced by siRNA, this reduced rosuvastatin-dependent CCR7 expression levels in RAW cells (Figures 5D and E). This indicates that SREBP-2 is a mediator of the effects of rosuvastatin treatment on CCR7 expression in macrophages. SREBP-2 nuclear expression was also up-regulated at the protein level in regressing plaques using immunohistochemistry, which further suggests a link between SREBP2 and CCR7 expression upon a reduction of non-HDL-cholesterol levels *in vivo* (Figure S2).

### Statins induce acetylation of histones H3 and H4 and recruit SREBP-2 and p300 to the promoter region of CCR7

Since histone acetylation is associated with gene activation, and SREBP-2 induces CCR7 expression, we examined whether changes in histone acetylation would affect CCR7 expression. Treatment with histone deacetylase inhibitor trichostatin A (TSA) resulted in the upregulation of CCR7 mRNA (Figure S3), suggesting that expression of CCR7 is repressed by HDACs and that SREBP-2 can overcome this inhibition, which is a common mechanism employed by transcription factors to induce gene expression.

**Figure 6:**
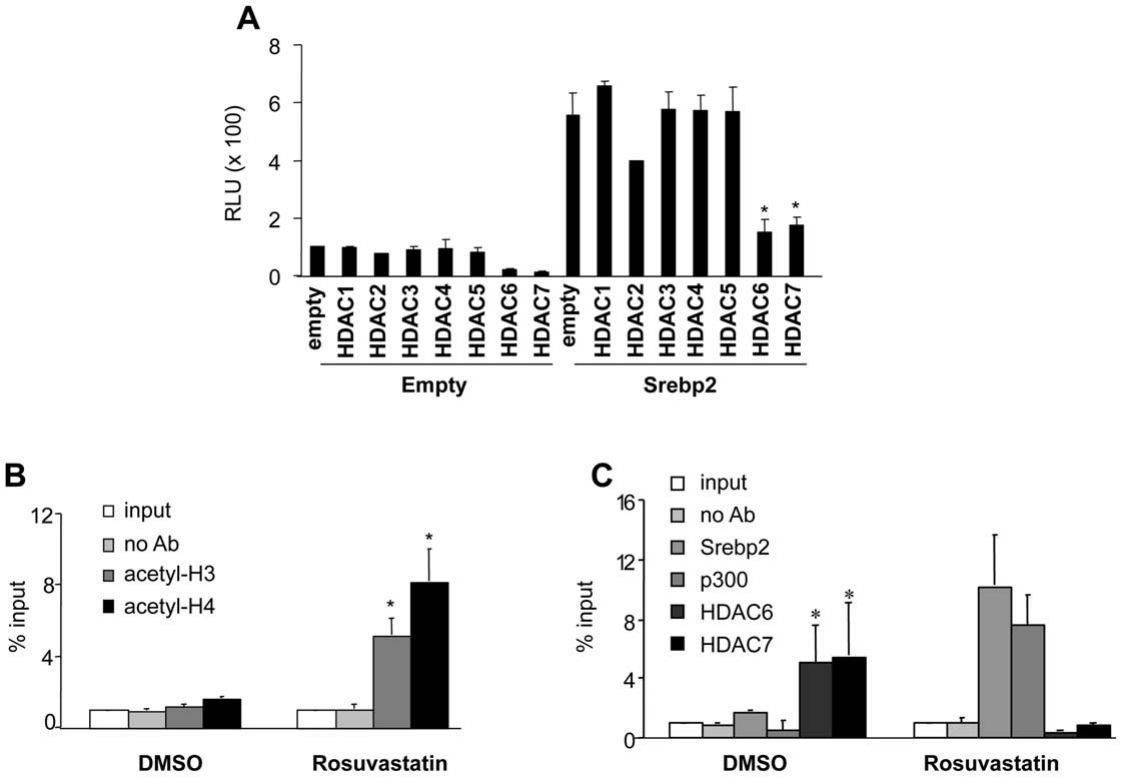
Statins induce acetylation of histones H3 and H4, with the dismissal of HDACs and recruitment of SREBP-2 and p300 to CCR7. Luciferase assays were performed in RAW264.7 cells co-transfected with HDAC expression plasmids, the SREBP-2 expression plasmid, and the CCR7 promoter-luciferase construct. B) RAW 264.7 cells were treated with 5 µM rosuvastatin and the recruitment of acetylated histone H3 and H4 at the promoter region of the CCR7 gene was assessed by ChIP assays (C). RAW264.7 cells were mock treated or treated with 5 µM rosuvastatin. ChIP assays of the CCR7 promoter were performed using antibodies against HDAC6, HDAC7, SREBP-2 and p300 in RAW264.7 cells either mock treated or treated with rosuvastatin. % Input values represent the mean ± SEM.

To test the impact of HDACs on the induction of CCR7 by SREBP-2, luciferase assays were performed in RAW264.7 cells co-transfected with expression plasmids for HDACs 1 through 8, along with an SREBP-2 expression plasmid, and the -850 bp CCR7 promoter-luciferase construct. Only HDAC6 and HDAC7 were shown to decrease the SREBP2-dependent CCR7 promoter activity (Figure 6A). This suggests that CCR7 promoter is repressed by HDAC6 and/or HDAC7, and that this repression in potentially relieved by SREBP-2.

To characterize the changes in chromatin organization and the factors recruited to the CCR7 regulatory region upon statin treatment, we performed chromatin immunoprecipitation assays (ChIP) for modified histones, SREBP-2 and various HDAC (HDAC6 & 7) and HAT (p300) coregulatory proteins. RAW264.7 cells were treated with rosuvastatin or DMSO, and acetylation levels of histone H3 and H4 at the promoter region of the CCR7 gene were assessed. Interestingly, rosuvastatin treatment promoted acetylation of histone H3 and H4, marks of transcriptional activation, at the promoter regions of the CCR7 gene (Figure 6B). We were able to demonstrate that rosuvastatin treatment of RAW264.7 cells led to the eviction of HDAC6 and HDAC7, and recruitment of SREBP-2 and p300 (Figure 6C). These data establish SREBP-2 as promoting CCR7 transcription, as well as suggest the mechanism by which statins influence this process.

## Discussion

Statins are potent inhibitors of cholesterol biosynthesis, and through the SREBP transcriptional factors they stimulate expression of many target genes, including that of the LDL receptor. In clinical trials, statins are beneficial in the primary and secondary prevention of coronary heart disease (reviewed in [1]). It has been suggested that there are a number of benefits to the arterial wall beyond LDL lowering[20]. Indeed, recent studies indicate that some of the cholesterol-independent or “pleiotropic” effects of statins involve improving endothelial function, enhancing the stability of atherosclerotic plaques, decreasing oxidative stress and inflammation, as well as inhibiting the thrombogenic response [22]. In this report, we describe another effect of statins, namely, their promotion of the depletion of CD68+ cells from plaques in a CCR7 dependent fashion.

We have previously reported that CCR7 was induced in plaque CD68+ cells and that it was functionally required for their emigration to regional and local lymph nodes when the hyperlipidemic plasma environment of the apoE^-/-^ mouse was normalized [12]. Here we have also found CCR7 function to be required for the effects of the statins on regression. There was some reduction in plasma cholesterol levels associated with statin treatment, though as noted earlier, they were still above the level that causes atherosclerosis progression [7], [17]. Still, this may have been sufficient to stimulate SREBP processing in the plaque CD68+ cells, or the effects of the statins on cellular sterol status in the plaque were more pronounced than the plasma levels of non-HDL cholesterol indicate. Certainly, in studies *in vitro*, the statins appeared to directly regulate CCR7 gene expression, not only via SREBPs (particularly SREBP-2) through the SRE, but also through chromatin remodeling.

Given the ability of statins to induce CD68+ cell emigration from mouse plaques, an obvious question is why these potent regulators of SRE-dependent transcription have not been more effective in promoting plaque regression in clinical trials. At best, after 2 years of intensive therapy reduced plaque volume by less than 1% when assessed by intravascular ultrasound (IVUS) [5]. Note that IVUS is sensitive to plaque size, not composition. If in the clinical studies there was enrichment of the plaque collagen as in the statin-treated mice, a real possibility is that IVUS would not have detected a change because it was the composition, not the size, of the plaques that was modified. Improved imaging techniques will be needed to resolve this issue in clinical studies.

Another possibility for the apparent discrepancy between the present and clinical results is that the SRE pathway remains more down-regulated in human CD68+ plaque cells, even with LDL lowering, and that an increase in HDL is necessary. This would be consistent with the meta-analysis that showed the optimal setting for clinical regression was when LDL was lowered and HDL was raised [23], and that even with lowered LDL, patients with low HDL were not protected from coronary artery disease risk

Our findings indicate that the mechanism of SREBP-2-dependent induction of CCR7 by rosuvastatin is mediated in part by the removal of the Class II HDACs, HDAC6 and HDAC7 from the CCR7 promoter. And although we only examined rosuvastatin in these mechanistic studies since it produced a more robust induction of CCR7 compared to atorvastatin, we would expect that atorvastatin would use the same mechanism of regulating CCR7 expression. Corepressor HDACs are thought to repress transcription by associating with gene promoters and are replaced by stimulating coactivator HATs (e.g. p300) for subsequent activation upon signal transduction, which is what we observe upon CCR7 induction by statins through SREBP-2. HDAC7 appears to be upregulated in mouse models of atherosclerosis [24], consistent with the very low expression of CCR7 in CD68+ cells from atherosclerotic plaques [12]. HDAC6 is largely a cytoplasm enzyme that acetylates and modulates alpha-tubulin and Hsp90, however a portion of HDAC6 also resides in the nucleus and has been shown to be bound to chromatin, deacetylase histone N-terminal tails, and repress gene expression (reviewed in [25]). Given the role of cytoplasmic HDAC6 in cell migration via changes in the cytoskeleton [26], it is tempting to speculate that dismissal of HDAC6 from nuclear chromatin upon statin treatment could increase cytoplasmic pools of HDAC6, thereby coupling the induction of CCR7 expression by statins with cell migration. Although HDAC7 mice are embryonic lethal [27], HADC6 deficient mice are viable and fertile [28], and it will be interesting to see in future studies if regression of atherosclerosis is impaired in HDAC6 deficient mice upon statin treatment.

Taken together, our findings indicate that statins, in addition to their traditionally considered effects on atherosclerosis by retarding plaque progression through lowering LDL-C, may also have additional clinical benefits by accelerating plaque regression through enhancing CCR7 expression and emigration of CD68+ macrophages [29]. Importantly, the beneficial effects would have been missed if changes in plaque size had been the sole criterion of success.

## Materials and Methods

### Experimental Design & Animals

The NYU School of Medicine Institutional Animal Care and Utilization Advisory Committee approved all animal procedures. Protocol Number: 081003-02 Title: Molecular Regulation of Atherosclerosis Regression; Approval date: 11/18/10. Four to five-weeks old apoE^-/-^ mice (n = 60) were weaned onto 0.15% cholesterol and 21% fat Western-type diet (WD; Research Diets, Inc) for 16 weeks to develop atherosclerotic lesions. Fifteen mice were sacrificed for baseline analysis and the remaining forty-five were divided into three groups: The first group continued on the western diet, the second and third groups, respectively, were fed western diets containing atorvastatin and rosuvastatin for 4 weeks at concentrations of 0.016 g per kg diet for rosuvastatin and 0.01 g per kg diet for atorvastatin. This yields 2.4 mg/kg mouse per day for rosuvastatin, and 1.5 mg/kg mouse per day for atorvastatin, assuming a 30 g mouse consuming 5 g food/day. The Ccr7^-/-^ mice [14] (from S. Lira, Mount Sinai School of Medicine, with permission from M. Lipp, Max Delbrück Center for Molecular Medicine) were crossed with apoE^-/-^mice to generate Ccr7^-/-^/apoE^-/-^ double-knockout mice. At 4 weeks of age, mice were placed on WD for 16 weeks, and then switched to either WD-containing atorvastatin or rosuvastatin at the concentrations described above for 4 weeks. At this point, analyses were performed as described below.

### Lipid and Lipoprotein Analyses

Blood samples were obtained from the retro-orbital plexus of the various strains of mice studied. Plasma total cholesterol levels were determined by colorimetric enzymatic assays that was adapted to 96-well plate formats (Infinity Total Cholesterol Reagent or Infinity Triglyceride Reagent, Sigma). Plasma HDL cholesterol was determined by precipitating non–HDL cholesterol (Wako Diagnostic) and then assaying the remaining HDL cholesterol by means of the Infinity Total Cholesterol Reagent.

### Labeling of Blood Monocytes

For selective labeling of Ly-6C^hi^ (CCR2 positive) and Ly-6C^low^ monocytes, we used the protocols as described in [19], [30]. Fluorescent beads in the plaque were counted in a blinded fashion.

### Tissue Processing

Mice were sacrificed and the portion of hearts containing the proximal aorta (aortic root) was collected and processed for frozen blocks as previously described [12]. Frozen serial sections were cut at 6-µm thickness and mounted on positively charged slides (Color Frost Plus; Fisher Scientific).

### Histology, Immunohistochemistry, and Morphometric Analyses

Every fifth slide from the serial sections was stained with CD68 antibody as previously described [31] and used for morphometric analysis and as a guiding slide for laser capture microdissection (see below). The intimal and CD68-immunostained areas were quantified by computer-aided morphometric analysis (Image Pro Plus 3.0 software; Media Cybernetics, Silver Spring, MD) performed on digitized microscopic images. In order to assess cholesteryl ester content, slides were stained with oil-red-O as previously described [32]. Collagen content was assessed by Sirius Red staining [33]. MCP-1 immunostaining was performed using a biotin-linked anti-mouse MCP-1 antibody (BioLegend- cat #505908,) and visualized using the VECTASTAIN® ABC-AP system coupled with the Vector Red substrate (Vector labs) and imaged under a Ziess confocal fluorescent microscope.

### Laser Capture Microdissection and RNA Extraction

For laser capture microdisssection (LCM), all reagents were maintained and all procedures were performed under RNase-free conditions. Tissue sections were stained with hematoxylin & eosin according to a quick staining protocol. Briefly, sections were fixed in 70% ethanol for 1 min, washed in H_2_O, stained with Mayer's hematoxylin (VWR Scientific) for 1 min, washed in H_2_O, incubated in PBS (to develop blue color) for 15 sec, washed in H_2_O, partially dehydrated in 70% followed by 95% ethanol, stained in eosin Y (VWR Scientific) for 5 sec, washed in 95% ethanol, and completely dehydrated in 100% ethanol (30 sec), xylene (30 sec) and xylene (5 min). After air-drying for 10 min, foam cells could be identified under a microscope and be verified by the CD68 staining on the guiding slides. Captured tissues from 3∼5 animals from the same treatment group and time point were pooled, and RNA was extracted using the QIAGEN micro RNA kit with on-column DNase I treatment following the manufacturer's instruction. The concentration of RNA was determined by the Ribogreen RNA Quantitation kit (Molecular Probes), and the RNA quality verified with the Agilent 2100 Bioanalyzer.

### Quantitative Real-Time (RT)-PCR

RNA abundances were determined by RT-PCR using 100pg of total RNA. Concentrations of total RNA were measured by spectrophotometry (Nanodrop ND-100 spectrophotometer from Biolabs). Reverse Transcriptase PCR was performed on samples using the iScript cDNA Synthesis kit (Bio Rad), per manufacturer's protocol.

The mRNA levels of CCR7, SREBP-1a, SREBP-1c and SREBP-2 were normalized against 18S rRNA. The primer and probe sequences used are the same as described either in the following references [34], [35] or were: SREBP1a: 5′-GCGCCATGGACGAGCTG-3′ and 5′-TTGGCACCTGGGCTGCT-3′; SREBP1c: 5′-GGAGCCATGGATTGCACATT-3′, and 5′-GCTTCCAGAGAGGAGGCCAG-3′; SREBP2: 5′-CCCTTGACTTCCTTGCTGCA-3′, and 5′-GCGTGAGTGTGGGCGAATC-3′. For the laser-captured CD68+ cell data, the results are from two independent samples, each one representing a pool of foam cell RNA from three animals.

### Cell Culture and Treatments

RAW264.7 cells were obtained from ATCC and maintained in Dulbecco's modified Eagle's medium with 10% FBS. In the time course experiment, cells were treated with serum-free media containing DMSO or one of the following: rosuvastatin (Astra-Zeneca); lovastatin (Sigma); atorvastatin (Pfizer). All statins were used at a final concentration of 5 µM. Cells were harvested for total RNA using Trizol (Sigma) according to the manufacturer's instructions at 1, 3, 6, and 24 hours post treatment. In the siRNA experiment, RAW264.7 cells were treated with 5 µM of rosuvastatin and harvested for total RNA using Trizol (Invitrogen) according to the manufacturer's instructions.

### Transfections and Luciferase Assays

Full length and mature forms of mouse SREBP-1a, -1c and -2 expression plasmids were kindly provided by Dr. Timothy Osborne (University of California, Irvine, California) [36]. siRNAs for SREBP-2 and Control siRNA were purchased from Thermo Fisher, and transfected according to the manufacturer's instructions. Seventy-two hours after transfection, luciferase activity was assessed and normalized for cotransfection efficiency by β-galactosidase activity. The endogenous gene expression was determined by qRT-PCR.

RAW264.7 cells were transfected by electroporation (Amaxa), using the manufacturer's instructions. COS7 cells were transfected with Lipofectamine 2000 (Invitrogen) as described in Rayner *et al* [37]. The CCR7-lucfierase reporter constructs have been previously described[15]. The mutations in the SRE were generated using the Multi-site-Quickchange kit (Stratagene) according to the manufacturer's protocol using the following oligonucleotides: forward 5′-CCC GAG CCT CAG CCT ATC TGT CCC CTC AGC A-3′; reverse 5′-TGC TGA GGG GAC AGA TAG GCT GAG GCT CGG G-3′. The mutations were confirmed by sequencing. In addition to the substitutions, a single nucleotide deletion was also introduced that further disrupted the SRE. At 24h post transfection, cells were lysed with Passive Lysis Buffer (Promega) as per the manufacturer's instructions. The Luciferase Reporter Assay System (Promega) and the LMax microplate reader luminometer were used to determine luciferase activity.

### Chromatin immunoprecipitation (ChIP) Assays

ChIP assays were performed as previously described [38]. RAW264.7 cells were plated 24 hours before the treatment of rosuvastatin for 1h. Then the cells were fixed with 1% formaldehyde for 10 min at 37°C to cross-link protein-DNA and protein-protein interactions within intact chromatin. The cross-linked chromatin was sonicated to shear chromatin fragments to 200–400 bps. Chromatin-protein complexes were immunoprecipitated with antibodies against acetyl histone H3 (Millipore; 06-599), and H4 (Millipore; 12-344), SREBP-2 (Santa Cruz Biotechnology; sc-13552), p300 (Santa Cruz Biotechnology; sc-584), HDAC6 and 7 (Cell Signaling Technology; 2162 and 2882). The salmon sperm DNA/protein A agarose was added. The antibody was excluded from the immunoprecipitation reaction as negative control. Samples were then washed, reverse cross-linked, purified, and subjected to Real-time PCR to assess the association of these transcription factors on the SRE-containing promoter region of the CCR7 gene.

### Statistics

All data are expressed as average ± SEM. For lesion morphometry, the number of animals in each condition is indicated in the figure legends. For RNA analysis, two pooled samples were used for each condition. PRISM software (GraphPad, San Diego) was used to analyze differences between samples by one-way ANOVA with the Bonferroni post-test for differences between selected pairs of samples. *P* values of <0.05 were considered significant.

## Supporting Information

Figure S1 **Organization of the CCR7 gene and location of predicted sterol response elements (SRE).** To identify potential binding sites for transcription factors that may be important in the regulation of CCR7 gene expression, 6 Kb of the human and mouse CCR7 upstream regulatory regions were analyzed by MatInspector (Bioinformatics. 2005; 21:2933-42). This revealed a conserved sterol responsive element (SRE), in both human and mouse CCR7.
(PDF)

Figure S2 **Expression of SREBP-2 in atherosclerotic plaques.** Aortic arches from 20-week western diet fed donor apoE-/- were transplanted into wild type (EKO-WT; regression conditions) (A) or apoE-/- (EKO-EKO; progression conditions) recipients. At 3 days post-transplant the grafts were harvested. Serial aortic cryosections were immunostained for SREBP-2. No staining above background is evident in the presence of the secondary antibody alone (not shown). The striped green background signal is due to autofluorescence from the internal elastic lamina from the medial layer of the artery.
(PDF)

Figure S3 **The histone deacetylase inhibitor trichostatin A (TSA) induces CCR7 mRNA expression.** RAW macrophages were incubated for 24h in medium with 1% FBS and DMSO vehicle or 20ng/ml TSA for 24 h. Transcripts were analyzed by real time Q-PCR. Values indicate expression of CCR7 normalized to cyclophilin and levels are presented as fold induction relative to the expression in DMSO-treated cells, which was arbitrarily set to 1.
(PDF)
