# Supplementary material for: Statins Promote the Regression of Atherosclerosis via Activation of the CCR7-Dependent Emigration Pathway in Macrophages
Source: PLoS One. 2011 Dec 6;6(12):e28534. doi: 10.1371/journal.pone.0028534 (PMC3232231; doi:10.1371/journal.pone.0028534)
Supplement: Figure S1 — Organization of the CCR7 gene and location of predicted sterol response elements (SRE). To identify potential binding sites for transcription factors that may be important in the regulation of CCR7 gene expression, 6 Kb of the human and mouse CCR7 upstream regulatory regions were analyzed by MatInspector (Bioinformatics. 2005; 21:2933-42). This revealed a conserved sterol responsive element (SRE), in both human and mouse CCR7. (PDF) [file pone.0028534.s001.pdf]

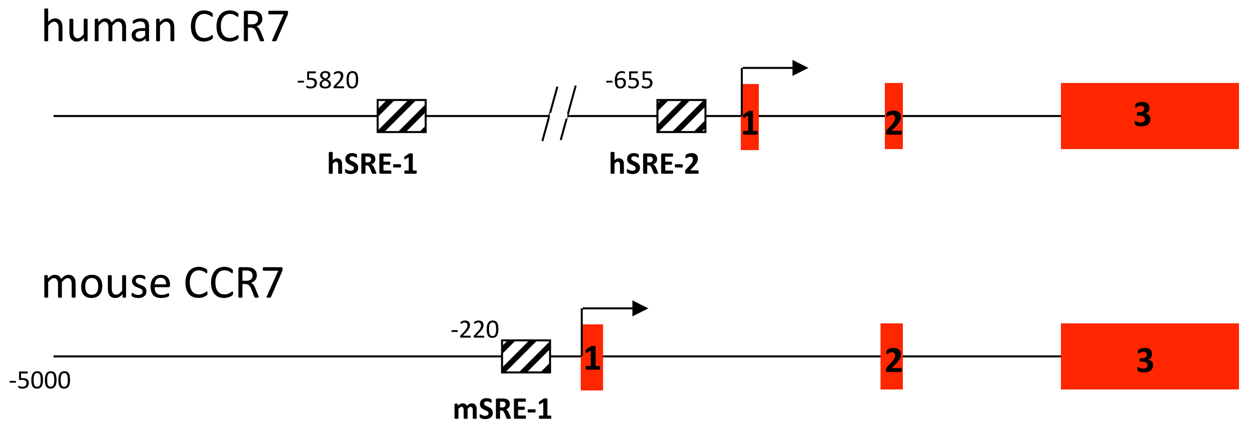

**hSRE-1**	ATCACCACAC
**hSRE-2**	ATCACCACAA
**mSRE-1**	CTCACCCCAC
consensus SRE	ATCACCCCAC

Supplementary Figure 1. Organization of the CCR7 gene and location of predicted sterol response elements (SRE)
To identify potential binding sites for transcription factors that may be important in the regulation of CCR7 gene expression, 6 Kb of the human and mouse CCR7 upstream regulatory regions were analyzed by MatInspector (Bioinformatics. 2005; 21:2933-42). This revealed a conserved sterol responsive element (SRE), in both human and mouse CCR7.
