# Supplementary material for: Statins Promote the Regression of Atherosclerosis via Activation of the CCR7-Dependent Emigration Pathway in Macrophages
Source: PLoS One. 2011 Dec 6;6(12):e28534. doi: 10.1371/journal.pone.0028534 (PMC3232231; doi:10.1371/journal.pone.0028534)
Supplement: Figure S2 — Expression of SREBP-2 in atherosclerotic plaques. Aortic arches from 20-week western diet fed donor apoE-/- were transplanted into wild type (EKO-WT; regression conditions) (A) or apoE-/- (EKO-EKO; progression conditions) recipients. At 3 days post-transplant the grafts were harvested. Serial aortic cryosections were immunostained for SREBP-2. No staining above background is evident in the presence of the secondary antibody alone (not shown). The striped green background signal is due to autofluorescence from the internal elastic lamina from the medial layer of the artery. (PDF) [file pone.0028534.s002.pdf]

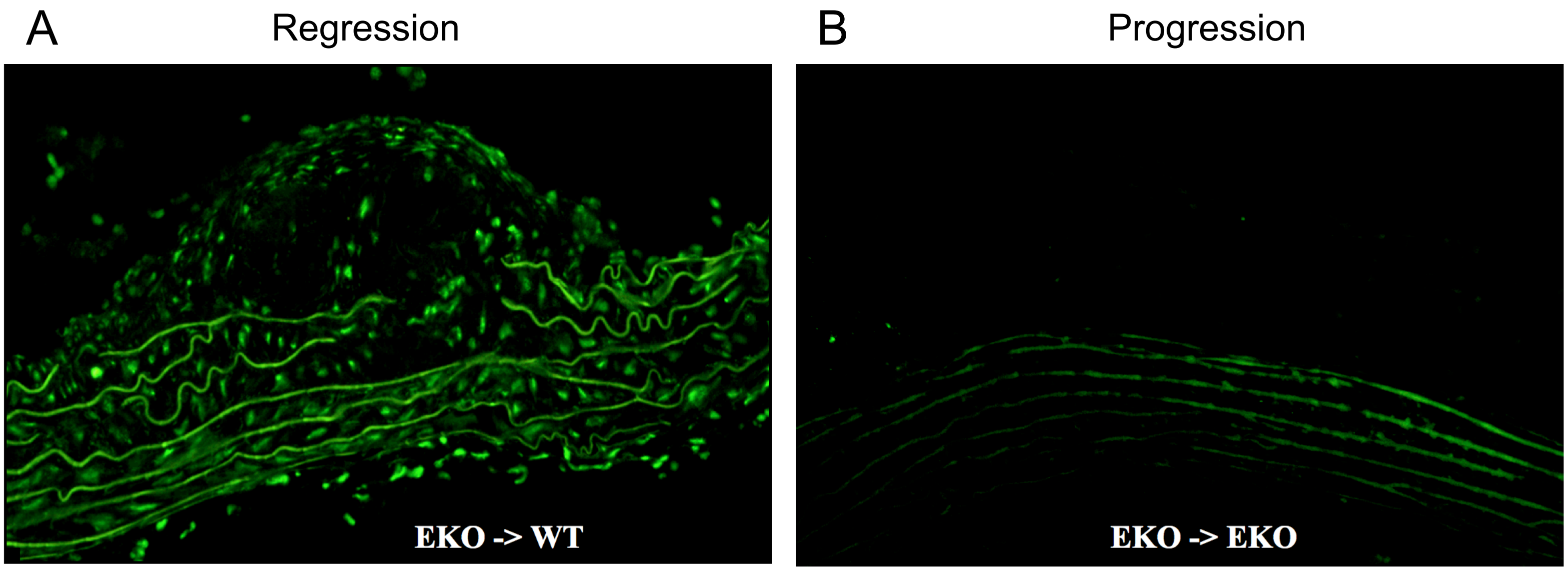

Supplemental Figure 2. Expression of SREBP-2 in atherosclerotic plaques. Aortic arches from 20-week western diet fed donor apoE-/- were transplanted into wild type (EKO-WT; regression conditions) (A) or apoE-/- (EKO-EKO; progression conditions) recipients. At 3 days post-transplant the grafts were harvested. Serial aortic cryosections were immunostained for SREBP-2. No staining above background is evident in the presence of the secondary antibody alone (not shown). The striped green background signal is due to autofluorescence from the internal elastic lamina from the medial layer of the artery.
