# Supplementary material for: Statins Promote the Regression of Atherosclerosis via Activation of the CCR7-Dependent Emigration Pathway in Macrophages
Source: PLoS One. 2011 Dec 6;6(12):e28534. doi: 10.1371/journal.pone.0028534 (PMC3232231; doi:10.1371/journal.pone.0028534)
Supplement: Figure S3 — The histone deacetylase inhibitor trichostatin A (TSA) induces CCR7 mRNA expression. RAW macrophages were incubated for 24h in medium with 1% FBS and DMSO vehicle or 20ng/ml TSA for 24 h. Transcripts were analyzed by real time Q-PCR. Values indicate expression of CCR7 normalized to cyclophilin and levels are presented as fold induction relative to the expression in DMSO-treated cells, which was arbitrarily set to 1. (PDF) [file pone.0028534.s003.pdf]

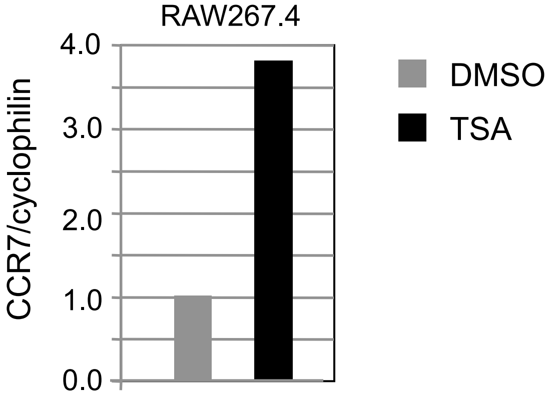

Supplementary Figure 3. The histone deacetylase inhibitor trichostatin A (TSA) induces CCR7 mRNA expression. RAW macrophages were incubated for 24h in medium with 1% FBS and DMSO vehicle or 20ng/ml TSA for 24 h. Transcripts were analyzed by real time Q-PCR. Values indicate expression of CCR7 normalized to cyclophilin and levels are presented as fold induction relative to the expression in DMSO-treated cells, which was arbitrarily set to 1.
